# Activating attachments enhances heart rate variability

**DOI:** 10.1371/journal.pone.0151747

**Published:** 2018-02-15

**Authors:** Richard A. Bryant, Thea Hutanamon

**Affiliations:** University of New South Wales, New South Wales, Australia; Leibniz Institute for Neurobiology, GERMANY

## Abstract

Although activating mental representations of attachment figures is beneficial for psychological health, there is a paucity of knowledge of the underlying mechanisms. We investigated how priming attachment figures may modulate parasympathetic stress response. Participants (N = 62) with varying degrees of attachment security underwent a cold pressor test, and then imagined an attachment or non-attachment figure. Heart rate variability was assessed throughout the study. Participants with low avoidant attachment levels displayed less negative affect and greater heart rate variability following the attachment prime than those who imagined the non-attachment prime. This beneficial effect of attachment priming was not observed in participants with high avoidant attachment levels. These findings highlight that activating attachment representations can enhance the parasympathetic stress response in people with secure attachment styles, and provides one explanation for the psychological benefits of attachment proximity.

## Introduction

Accessing social supports is one of the key strategies humans use to manage stressful experiences [1]. Attachment theories propose that from the cradle people become aware that one requires the nurturing of care-givers during times of need; this pattern subsequently develops such that during periods of threat, one tends to seek out attachment figures to provide support and care [2]. Supporting this view is much evidence that having access to attachment figures reduces the impact of stressful events, such as physical pain [3–5]. Further, when mental representations of attachment figures are activated people enjoy reduced stress reactions, including reduced attentional bias to threat [6, 7], noradrenergic response [8], and pain-related neural activation [9].

These effects are moderated, however, by a person’s attachment style. Attachment theories posit that early inconsistent attachment experiences result in poorly developed mental representations of secure attachments, and so during stressful experiences such representations do not confer optimal benefits [10]. Insecure attachment styles comprise two types. Some people are anxiously attached, and are characterized by excessively seeking attachments because they fear they will not be available; such people typically *hyperactivate* their attachment systems in times of threat because they rely heavily on attachment figures. In contrast, others are avoidantly attached and dismiss attachments because they have learnt from past experiences that they are unavailable; these people *hypoactivate* their attachment systems, and respond to threat by distancing themselves from others and using other coping strategies [10]. Experimental studies have shown that during threat avoidantly attached individuals inhibit proximity-seeking behaviour [6] and are less likely to activate attachment representations [11].

Despite this evidence, there is inadequate understanding of the exact mechanisms by which attachment activation influences the observed outcomes. Relevant to this issue is polyvagal theory, which posits that social behavior requires inhibition of systems required for threat-related responses, such as the fight or flight response [12]. It is argued that the vagus nerve, which rapidly modulates heart rate and other visceral organs to allow the organism to engage or disengage from the environment, modulates sympathetic arousal during threat response but then facilitates parasympathetic response when threat is absent to allow prosocial behaviors [13]. The core index of vagal activity is heart rate variability (HRV), which reflects rhythmic oscillations of heart rate that occur synchronously with breathing. To date there is no evidence regarding the impact of activating attachment representations on HRV. Accordingly, this study assessed the impact of attachment representations on HRV following a stressor. We hypothesized that activating attachments would promote vagal activity, and thereby result in enhanced HRV following the stressor relative to activation of a non-attachment representation.

## Methods

### Participants

Participants (*N* = 62) were first-year psychology students (39 females, 23 males) of mean age 19.17 years (*SD* = 2.01) at the University of New South Wales who participated in return for course credit. Participants were instructed to either Attachment (n = 31) or Non-Attachment (n = 31) conditions. Participants were excluded if they were either undertaking heart medications, and have severe or very severe depression and anxiety scores on the Depression, Anxiety and Stress Scale (DASS) [14]. The study was approved by the University of New South Wales School of Psychology Ethics Review Committee, and all participants gave written informed consent (which was approved by the Ethics Committee). The raw data are available as supporting information; see S1 Data.

### Heart rate variability

Heart rate was recorded via a standard three-electrode setup, using a Lead II configuration connected to an ADInstruments System (Colardo Springs,USA; http://www.adinstruments.com). Electrodes were attached on the back of the participant’s neck, and right and left clavicles. The electrocardiogram (ECG) signal was sampled at 1000 Hz, with a high-pass filter of 0.5 Hz [15] across three five-minute recording periods. The range of cyclic measurement of HR was at 100mv, and ECG signals were 0.3Hz-100Hz bandpass-filtered. To obtain frequency-domain indices of HRV, we used autoregressive estimates of high-frequency power (HRV; 0.15–0.40 ms^2^/Hz) in power percentage, which provides an estimate of parasympathetic nervous system activity [15]. This protocol adheres with the recommendations of the Task Force of the European Society of Cardiology and the North American Society of Pacing Electrophysiology [15].

### Procedure

**Fig 1:**
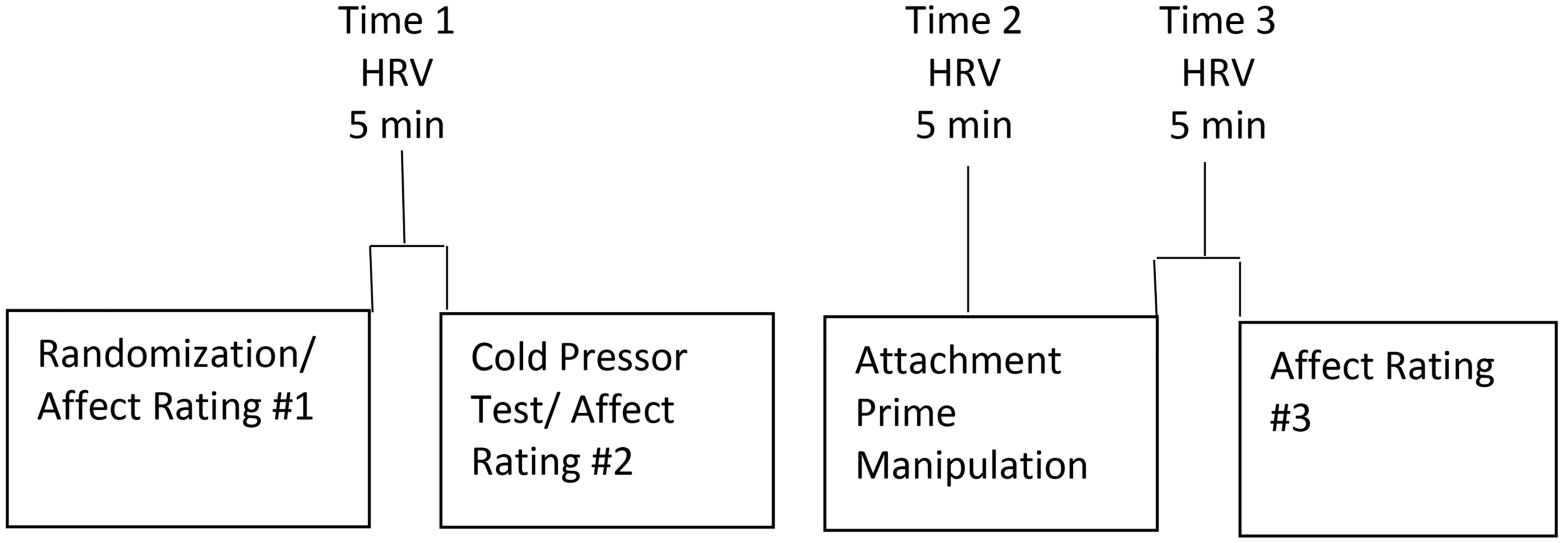
Flowchart of experimental session.

Prior to the experimental session, participants were instructed not to exercise for 24 hours or consume caffeine for three hours prior to the study. Participants were initially randomized to either the Attachment (n = 31) or non-Attachment (n = 31) prime conditions (see Fig 1 for experimental flowchart). Participants initially completed the DASS, and then were asked to nominate either attachment or non-attachment figures. Participants then completed the negative scale of the Positive Affect and Negative Affect Schedule (PANAS) to measure participants’ mood (Watson, Clark & Tellegen, 1988), the Short Form of the International Physical Activity Questionnaire (IPAQ) to assess physical activity in the seven days prior to the session [16], and body mass index (BMI) was assessed. Participants also completed the Experiences in Close Relationships scale (ECR) [17], which is a 36-item self-report scale that comprises two subscales: anxious attachment and avoidant attachment, with each item is scored on a 7-point Likert scale. We determined high and low scorers on the anxious attachment and avoidant attachment scales, respectively by calculating medium splits on each subscale (52 on the Avoidant Attachment and 58 on Anxious Attachment). Participants also completed the Vividness of Visual Imagery Questionnaire (VVIQ), which consists of 16 items that involves imaging a specific person or scene [18]. Participants were asked to image each scene and then rate on a 5-point Likert scale (1 = *No image at all*, *you only know that you are thinking about the object*, 5 = *Perfectly clear and vivid as normal vision*). The VVIQ was used as a measure of individual differences in vividness in visual imagery because imaging the attachment figure was employed as the key manipulation of the study.

Electrodes were then attached, and an initial 5-min baseline HRV recording was obtained (Time 1). Participants were then administered a modified Socially Evaluated Cold Pressor Test (SECPT), which has repeatedly been shown to elicit strong stress responses [19]. Participants were instructed to place their left forearm in a bucket of ice water (3°C) for a period of 90 sec. To increase their stress response, they were instructed that they would be evaluated by experts on their performance via a videorecording system that was operating throughout the cold pressor test [20]. In a variation to the standard SEPCT, although participants received these instructions during the videoing of the cold pressor test, their responses were not actually evaluated and they did not receive direct feedback about their performance.

Immediately following removal of the arm from the ice water, participants were instructed to imagine the attachment or non-attachment figure they had previously nominated. Participants were told to imagine that person for a period of 5 min. Participant’s HRV was measured throughout the 5 min when they mentally visualised the attachment or non-attachment figure (Time 2). Participants were instructed to stop imagining the figure, and the PANAS was administered again. HRV was then recorded for another 5 minutes (Time 3). A final PANAS was administered, the electrodes were then removed, and participants were debriefed.

## Results

### Participant characteristics

Planned comparisons indicated that there were no statistically significant differences in age, BMI, physical activity levels, DASS scores, imagery capacity, or attachment style scores between participants in the Attachment and Non-Attachment conditions (see Table 1). There was no difference between conditions in terms of gender, with females comprising 67% of the Attachment sample and 60% of the Non-Attachment (χ^2^ = 0.59, *p* = .79).

**Table 1:** Participant characteristics.

	Attachment Prime (*n = 31;50%)*	Non-Attachment Prime (*n = 31;50%)*	*t (p)*
	*M (SD)*	*M (SD)*	
Age	19.50 (2.11)	19.10 (1.81)	.78 (.43)
BMI	20.58 (6.18)	21.81 (3.17)	.10 (.26)
Physical Activity	2.13 (.57)	2.28 (.53)	.10 (.32)
DASS Depression	4.58 (4.33)	6.19 (5.30)	1.31(.27)
DASS Anxiety	4.00 (4.16)	4.97 (4.09)	.92 (.80)
DASS Stress	10.32 (6.99)	10.83 (6.77)	.30 (.94)
Avoidant Attachment Score	52.68 (19.06)	54.68 (16.89)	.44 (.71)
Anxiety Attachment Score	60.00 (14.11)	54.87 (16.87)	-1.30 (.20)
VVIQ	59.10 (9.68)	55.77 (9.27)	-1.38 (.65)

*Note*. Standard deviations appear in parentheses.

### Negative affect ratings

Separate 2 (Attachment Condition) x 3 (Assessment Period) x 2 (AttachmentStyle) repeated measures analyses of variance (ANOVAs) of negative affect scores (based on PANAS scores) were conducted for participants with high and low attachment characteristics (i.e. high and low scores on anxious and avoidant attachment scores, respectively) (see Table 2). In terms of avoidant attachment, a 2 (Attachment Condition) x 3 (Assessment Period) x 2 (Avoidant Attachment) repeated measures ANOVA of PANAS scores indicated a main effect for Assessment Period [*F* (2, 55) = 41.33, *p* = .000], with participants reporting more negative affect following the stressor relative both to baseline and the follow-up period. There was a significant three-way Assessment Period x Attachment Condition x Avoidant Attachment interaction effect [*F* (2, 55) = 3.85 *p* = .03]. To delineate the three-way interaction, two-way ANOVAs were conducted for participants with high and low avoidant attachment, respectively. In terms of those with low avoidant attachment, there was a main effect for Assessment Period [*F* (2, 26) = 20.39, *p* = .000] and a significant Attachment Condition x Assessment Period interaction effect [*F* (2, 26) = 3.69, *p* = .04]. Participants with low avoidant attachment who received the attachment prime had less negative affect following the prime than those with the non-attachment prime. A comparable ANOVA for high avoidant participants indicated a comparable main effect for Assessment Period [*F* (2, 28) = 21.47, *p* = .000] but no interaction effect. In terms of anxious attachment, a 2 (Attachment Condition) x 3 (Assessment Period) x 2 (Anxious Attachment) repeated measures ANOVA of PANAS scores indicated a main effect for Assessment Period [*F* (2, 55) = 51.86, *p* = .000], with participants reporting more negative affect following the stressor relative both to baseline and the follow-up period. There was a significant three-way Assessment Period x Attachment Condition x Anxious Attachment interaction effect [*F* (2, 55) = 4.47, *p* = .02]. In terms of those with low anxious attachment, there was a main effect for Assessment Period [*F* (2, 28) = 24.65, *p* = .000] and a significant Attachment Condition x Assessment Period interaction effect [*F* (2, 28) = 5.23, *p* = .01]. Participants with low anxious attachment who received the attachment prime had less negative affect following the prime than those with the non-attachment prime. A comparable ANOVA for high anxious attachment participants indicated a comparable main effect for Assessment Period [*F* (2, 26) = 27.42, *p* = .000] but no interaction effect.

**Table 2:** Mean negative affective scores.

	Avoidant Attachment			
	High		Low	
	Attachment	Non-Attachment	Attachment	Non-Attachment
Time 1	6.60 (2.06)	7.25 (1.73)	5.87 (1.06)	6.29 (1.64)
Time 2	11.00 (4.26)	10.50 (4.21)	7.60 (1.96)	10.79 (3.70)
Time 3	6.53 (1.96)	5.81 (1.28)	5.60 (0.91)	6.07 (1.73)
	Anxious Attachment			
	High		Low	
	Attachment	Non-Attachment	Attachment	Non-Attachment
Time 1	6.90 (2.18)	7.11 (1.94)	5.90 (1.25)	6.28 (1.19)
Time 2	12.30 (3.30)	10.52 (4.31)	7.80 (2.88)	10.82 (3.31)
Time 3	6.10 (1.29)	6.05 (1.68)	6.05 (1.73)	5.73 (1.10)

*Note*. Standard deviations appear in parentheses.

### Heart rate variability

**Fig 2:**
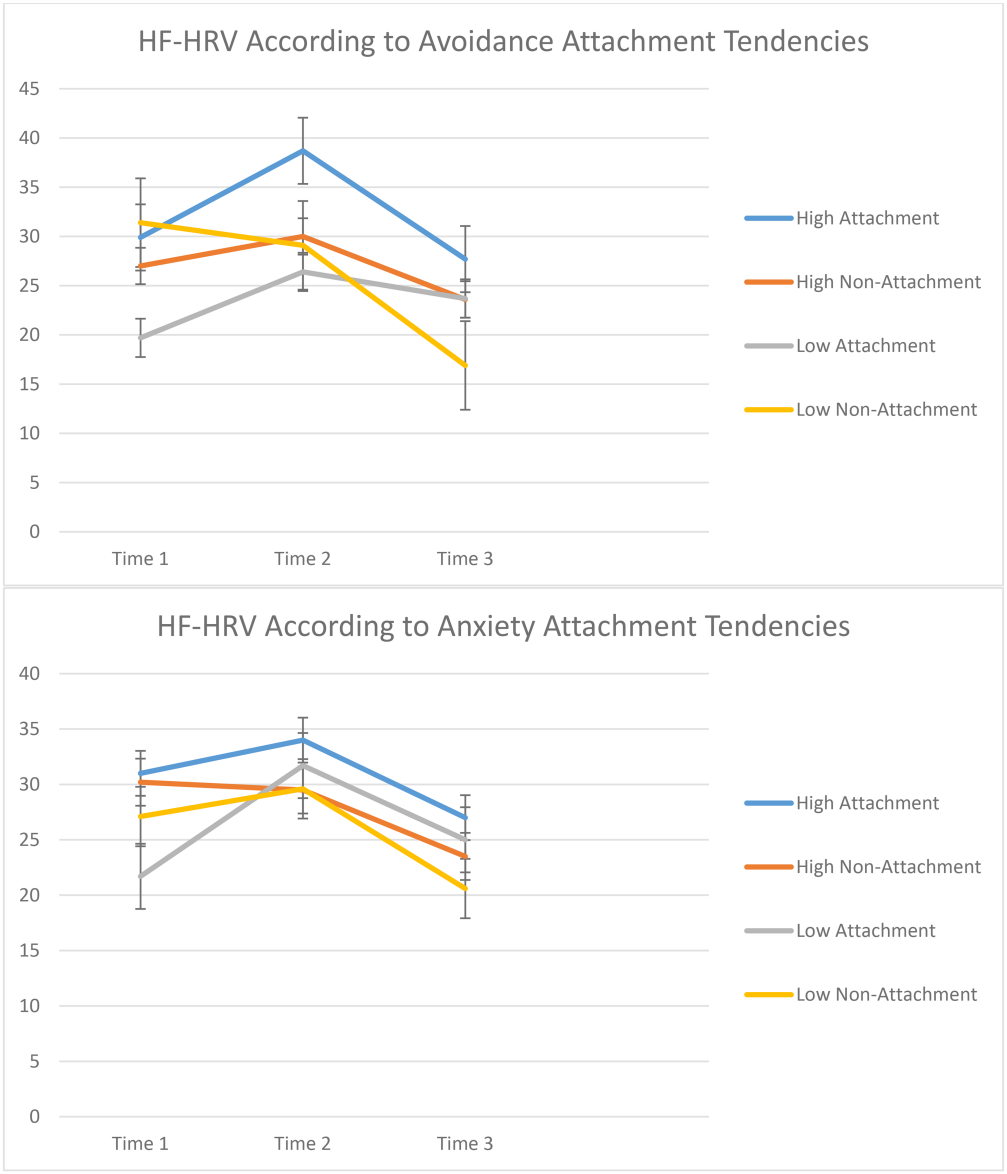
Average high frequency-heart rate variability across the three assessment periods for attachment prime and non-attachment prime groups. Error bars indicate standard error of the mean (SEM). High Attachment = High levels on relevant attachment style in the attachment prime condition. High Non-Attachment = High levels on relevant attachment style in the non-attachment prime condition. Low Attachment = Low levels on relevant attachment style in the attachment prime condition. Low Non-Attachment = Low levels on relevant attachment style in the non-attachment prime condition.

The primary outcome was HRV during (a) 5-minute baseline, (b) 5-minute post SECPT/Attachment Prime, and (c) 5-minute rest phase (see Table 3). Separate 2 (Attachment Condition) x 3 (Assessment Period) x 2 (Attachment Style) repeated measures ANOVAs of HRV were conducted for participants with high and low anxious and avoidant attachment scores, respectively (Fig 2). In terms of avoidant attachment, a 2 (Attachment Condition) x 3 (Assessment Period) x 2 (Avoidant Attachment) repeated measures ANOVA of HRV indicated a main effect for Assessment Period [*F* (2, 55) = 7.75, *p* = .001], with participants reporting greater HRV during the stressor relative to baseline, and reduced HRV in the follow-up period relative to during the stressor. Additionally, there was a significant three-way Assessment Period x Attachment Condition x Avoidant Attachment interaction effect [*F* (2, 55) = 6.59 *p* = .003]. To delineate the three-way interaction, two-way ANOVAs were conducted for participants with high and low avoidant attachment, respectively. In terms of those with low avoidant attachment, there was a main effect for Assessment Period [2, 26) = 3.93, *p* = .03] and a significant Attachment Condition x Assessment Period interaction effect [*F*(2, 26) = 8.86, *p* = .001]. Specifically, the attachment prime did not impact on participants with high avoidant attachment scores; in contrast, for those with low avoidant attachment scores thinking of an attachment figure resulted in increased HRV during the follow-up period relative to those who thought of a non-attachment figure [*p* = .001]. In terms of those with high avoidant attachment, there was a main effect for Assessment Period [2, 28) = 6.41, *p* = .05], with participants displaying greater HRV during the stressor relative to baseline (*p* = .05), and reducing during the follow-up relative to during the stressor (*p* = .001).

**Table 3:** Mean high frequency heart rate variability values.

	Avoidant Attachment			
	High		Low	
	Attachment	Non-Attachment	Attachment	Non-Attachment
Time 1	29.89 (16.38)	27.04 (15.17)	19.68 (14.41)	31.38 (16.36)
Time 2	38.70 (15.72)	29.90 (16.30)	26.35 (16.26)	29.11 (13.88)
Time 3	27.72 (13.38)	23.68 (16.83)	23.68 (16.83)	16.91 (9.66)
	Anxious Attachment			
	High		Low	
	Attachment	Non-Attachment	Attachment	Non-Attachment
Time 1	31.05 (21.15)	30.19 (17.96)	21.66 (12.21)	27.11 (10.97)
Time 2	34.16 (11.68)	34.16 (11.68)	31.70 (19.25)	29.64 (9.75)
Time 3	26.81 (18.21)	23.53 (14.75)	25.15 (13.75)	20.57 (8.44)

*Note*. Standard deviations appear in parentheses.

Parallel analyses that focused on people with high and low anxious attachment styles, respectively, found significant main effects for Assessment Period. For high anxious attachment participants [*F*(2, 26) = 7.73, *p* = .03], participants had lower HRV in the follow-up period relative to baseline (*p* = .03) and during the stressor (*p* = .008). For low anxious attachment participants [*F*(2, 28) = 3.29, *p* = .05], participants had greater HRV during the stressor relative to baseline (*p* = .01), and lower HRV during the follow-up relative to baseline (*p* = .02). All behavioral and heart rate variability data are presented in Supplementary Information (S1).

## Discussion

The major finding of this study was that participants with low avoidant attachment who imagined an attachment figure displayed greater vagal tone in the subsequent period than those who imagined someone who was not an attachment figure. This pattern was underscored by the comparable finding that attachment priming resulted in less reported negative affect in participants with low avoidant attachment relative to the non-attachment primes. The current findings accord with evidence that social support reduces physiological pain experience [4, 5], viewing a loved one’s photograph attenuates pain experience [21], and that viewing images of one’s partner is associated with less pain-related neural activation [9].

According to polyvagal theory [12, 13], the evolutionarily-driven reaction to threat is to activate the sympathetic nervous system, which readies the organism to react to the threat. This proposition holds that once the threat has abated, it is adaptive for the organism to initiate parasympathetic responses so that prosocial behaviors can be implemented. Extending polyvagal theory to attachment theory, it is possible that activation of mental representations of an attachment figure promoted HRV because this priming inhibited threat-related fight/flight responses, and increased parasympathetic activity via the myelinated vagus, which in turn triggered vagal tone response.

The beneficial effect of priming an attachment figure was evident in those with low avoidant attachment style but not in those with high avoidant attachment style. One previous report found that diminished resting HRV was associated with avoidant attachment style [22]. Attachment theory posits that people with avoidant attachment tendencies hypoactivate attachment systems during stress because of previous experiences of insecure attachment [23]; convergent evidence indicates that avoidantly attached people distance themselves from others during stress [6, 24, 25]. Further, priming avoidantly attached people with attachment figures fails to elicit the positive benefits afforded to securely attached people [26]. It seems that avoidantly attached tendencies were not associated with increased capacity to return to homeostasis when primed with an attachment figure because these participants are not able to utilize this system in response to stress. In this context it is noteworthy that an early study found that avoidantly attached individuals who used deactivating strategies when directed to think about attachment memories showed increases in skin conductance response, possibly suggesting conflict between such strategies and the confrontation of attachment [27].

We recognize some methodological limitations of these studies. First, our sample sizes were relatively small which precluded consideration of differential impact of sex, which has been shown to moderate stress response [28]. Second, we did not include a condition that involved a generic positive prime, which would have allowed us to discern between the effects of attachment and positive priming generally. It is worth noting, however, that previous studies have found that attachment primes confer greater psychological benefits than generic positive primes [29, 30]. Third, we could not directly index the extent to which participants continued to imagine the nominated figure throughout the acquisition of HRV; future studies could provide experimentally generated stimuli to provide a more direct reminder of the prime to increase the likelihood that it is available throughout the critical experimental period.

These findings provide some evidence for a biologically fundamental basis for attachment theory. For people with low avoidant attachment the availability of attachment figures appear to enhance parasympathetic responses in ways that dampen the experience of stress. This benefit does not appear available to those with insecure attachments. From an applied perspective, these results point to the potential of activating attachment representations in the aftermath of stress to facilitate adaptive response to the stressor.

## Supporting information

S1 Data (SAV)
